# Role of Multiparametric MRI in the Preoperative Evaluation of Endometrial Carcinoma: A Cross-Sectional Study

**DOI:** 10.7759/cureus.65058

**Published:** 2024-07-21

**Authors:** Saroj Kumar Pati, Kingshuk Mondal, Narendra Kuber Bodhey, Nilaj Bagde, Rakesh K Gupta, Arvind Shukla

**Affiliations:** 1 Radiodiagnosis, All India Institute of Medical Sciences, Raipur, Raipur, IND; 2 Obstetrics and Gynaecology, All India Institute of Medical Sciences, Raipur, Raipur, IND; 3 Pathology and Laboratory Medicine, All India Institute of Medical Sciences, Raipur, Raipur, IND; 4 Community and Family Medicine, All India Institute of Medical Sciences, Raipur, Raipur, IND

**Keywords:** diffusion tensor imaging, dynamic contrast-enhanced imaging, diffusion-weighted imaging, multiparametric mri, endometrial carcinoma

## Abstract

Background

Endometrial carcinoma (EC) is a major global concern in females throughout the world with increasing incidence in India. Hence, early detection and prompt intervention will reduce morbidity and mortality associated with it. Multiple studies showed a promising role of multiparametric magnetic resonance imaging (mpMRI) in the evaluation and early detection of the disease. In view of the paucity of such studies in the Indian population, we assessed the role of mpMRI in the evaluation of EC by utilizing a 3T MR scanner.

Objectives

To assess the efficacy of mpMRI in detecting myometrial invasion and locoregional staging in suspected or diagnosed cases of EC.

Materials and methods

Nineteen cases of EC with mpMRI were included in the study, and 15 of these underwent surgicopathological staging. The preoperative staging was done using the International Federation of Gynecology and Obstetrics (FIGO) 2009 staging system based on mpMRI findings and compared with postoperative FIGO staging. All the data were compiled in a Microsoft Excel (Microsoft® Corp., Redmond, WA) file and analyzed in Statistical Product and Service Solutions (SPSS, version 21.0; IBM SPSS Statistics for Windows, Armonk, NY) using appropriate tools.

Results

In our study, EC was commonly seen in more than 50-year females with a predominant complaint being postmenopausal bleeding. EC most commonly appeared heterogeneously hyperintense on T2-weighted sequence (T2W) and areas of diffusion restriction on diffusion-weighted imaging (DWI) in all cases. Dynamic contrast-enhanced (DCE) MRI (DCE-MRI) showed mild heterogeneous enhancement in all phases with better delineation of adjacent myometrial infiltration in the equilibrium phase. Diffusion tensor imaging (DTI) parameters had significantly lower values in involved myometrium vis-a-vis uninvolved myometrium. A statistically significant correlation was seen between preoperative mpMRI FIGO staging utilizing T2W, DWI, DCE-MRI, and DTI with surgicopathological FIGO staging.

Conclusion

mpMRI, particularly T2W, DWI, DCE-MRI, and DTI, yields a significant correlation between MR imaging and histopathological findings in assessing myometrial infiltration and thereby could be helpful in preoperative staging and extent of lymph-nodal dissection.

## Introduction

Endometrial carcinoma (EC) constitutes 3.7% of all malignancies occurring in females in India [1]. A rising trend particularly in urban areas is observed due to changing lifestyles and reproductive profiles [2]. The prognosis of EC depends on several factors, including depth of myometrial infiltration, lymphovascular invasion, histological grade, and nodal status [3]. Based on the depth of myometrial invasion, stage I EC is further classified into IA if no or less than 50% and IB if more than or equal to 50% of myometrium involved as per International Federation of Gynecology and Obstetrics (FIGO) 2009 staging [4]. Stage IB EC was seen to be more associated with lymph-nodal spread and therefore had higher chances of recurrence [3]. Hence, its early diagnosis not only improves the survival rate but also reduces its socioeconomic impact.

Multiparametric magnetic resonance imaging (mpMRI) could be an excellent non-invasive diagnostic tool to delineate myometrial invasion as per recent studies [5-9]. On dynamic contrast-enhanced MRI (DCE-MRI), EC enhances earlier than normal endometrium, which aids in detecting very small tumors confined to the endometrium. Normal myometrium on the other hand enhances more as compared to a relatively hypoenhancing tumor. This differential contrast is seen to be maximal at 50-120 seconds after intravenous administration of contrast medium. This is the most important phase for accurate assessment of the depth of myometrial invasion [9]. Studies revealed DCE-MRI, when read together with T2-weighted images increases the diagnostic ac­curacy up to 98% for assessing myometrial invasion [5-10]. In view of the paucity of such studies in the Indian population, we attempted to assess the myometrial invasion in endometrial cancer by mpMRI using 3T MRI in a suspected or diagnosed case of EC in this study.

## Materials and methods

The present cross-sectional study was performed in the Department of Radiodiagnosis, All India Institute of Medical Sciences (AIIMS), Raipur, Chhattisgarh, India, between September 2019 and July 2021 after obtaining ethical clearance from institute ethical committee (letter no: 778/IEC-AIIMSRPR/2019, dated 23/09/2019). Human subjects were involved in this research project in compliance with the Helsinki Declaration of 1964, its subsequent amendments, and related ethical standards, as well as the institute's ethical guidelines. Informed and written consent were obtained from all study participants.

A total of 22 patients with suspected/diagnosed cases of EC underwent mpMRI based on inclusion and exclusion criteria. Inclusion criteria were all suspected or diagnosed cases of EC with age ≥ 18 years. Patients with contraindications for MRI or with malignant neoplasms other than endometrial carcinoma were excluded from the study. Nineteen patients were found to have biopsy-proven EC and hence included in the study. However, 15 cases underwent surgicopathological staging as four patients did not undergo surgery for various reasons. Thus, 15 patients only could be analyzed for FIGO staging.

After proper instructions, participants were subjected to pelvic mpMRI in Discovery MR750w GEM (3.0 T MRI, GE, Chicago, IL) MRI scanner. They were placed in a supine position with a pelvic-phased array coil in place, and mpMRI was performed using the following protocol.

Magnetic Resonance Imaging (MRI) Protocol

Conventional sequences: T1-weighted sequence (T1W) in axial oblique and T2-weighted sequence (T2W) in axial, sagittal, and coronal oblique planes.

Diffusion-Weighted Imaging (DWI)

In the sagittal oblique and/or axial oblique plane, two b values (e.g., b = 500 and b = 800) were acquired. The apparent diffusion coefficient (ADC) map was calculated.

DCE-MRI

DCE-MRI images were acquired with a three-dimensional gradient-echo T1W fat-saturated sequence after the injection of 0.1 mmol/kg of gadolinium at a rate of 2 mL/s. Images were obtained at 0 seconds, 30 seconds, and 120 seconds after injection. A delayed sequence was acquired on the axial oblique at 4 min after injection.

Diffusion Tensor Imaging (DTI)

DTI data were acquired in the axial plane using a spin echo-based, single-shot echo-planar imaging sequence. Fractional anisotropy (FA), mean diffusivity (MD), and axial diffusivity (AD) maps were generated using corresponding DTI parameters.

Image and Data Analysis

Both conventional and mpMRI images were analyzed by a single radiologist with 10 years of experience in abdominal imaging blinded to both clinical and histopathological findings. By comparing mpMRI interpretation with the surgicopathological results, we analyzed its diagnostic yield in assessing FIGO staging particularly myometrial invasion.

Data were compiled in MS Excel (Microsoft® Corp., Redmond, WA) and analyzed using Statistical Product and Service Solutions (SPSS, ver 21.0; IBM SPSS Statistics for Windows, Armonk, NY). The data on categorical variables were shown as n (% of cases), and the data on continuous variables were presented as mean ± standard deviation (SD). An appropriate statistical tool was used for the comparison of data. P-value ≤0.05 was considered statistically significant. We attempted to reach a cut-off value to discriminate involved myometrium from normal myometrium based on the receiver operating characteristic (ROC) curve.

## Results

Out of 19 cases studied, the mean age of patients was 55.6 ± 9.8 years with the most common complaint being postmenopausal bleeding (78.9%), followed by pain in the lower abdomen (15.8%) in our study population. Three patients (15.8%) were nulliparous, out of which two had a clinical history of infertility, while the rest (84.2%) were multiparous. Based on the histopathology report, out of the 19 cases, 15 (78.9%) had endometrioid carcinoma, three (15.8%) had serous endometrial carcinoma, and one (5.3%) had carcinosarcoma (Table 1).

**Table 1:** Clinico-pathological profile of the study population (n=19)

Parameters	No. of cases (percentage)
Age	
≤ 50 years	5 (26.3)
>50 years	14 (73.7)
Clinical history	
Postmenopausal bleeding	15 (78.9)
Pain in the lower abdomen	3 (15.8)
Burning micturition	2 (10.5)
Heavy menstrual bleeding	2 (10.5)
Infertility	2 (10.5)
Loss of appetite	1 (5.3)
Parity	
Nulliparous	3 (15.8)
Multiparous	16 (84.2)
Histopathology	
Endometrioid carcinoma	15 (78.9)
Serous endometrial carcinoma	3 (15.8)
Carcinosarcoma	1 (5.3)

MRI findings

Among 19 EC studied on T2W images, EC was mostly heterogeneously hyperintense, followed by iso- to hyperintense in signal intensity as compared to normal myometrium. The most common appearance on T1W was iso- to hypointense, followed by isointense (Table 2).

**Table 2:** MRI findings in the study population (n=19) MRI = magnetic resonance imaging

Parameters	No. of cases (percentage)
MRI findings	
On T2W	
Heterogeneously hyperintense	13 (68.4)
Hyperintense	2 (10.5)
Iso to hyperintense	4 (21.1)
On T1W	
Hypointense	2 (10.5)
Isointense	7 (36.8)
Iso- to hypointense	10 (52.6)
Diffusion-Weighted MRI	
Areas of diffusion restriction	19 (100.0)
Dynamic contrast-enhanced MRI	
Present	18 (94.7)
Absent	1 (5.3)
Myometrial invasion	
<50%	9 (47.4)
>50%	6 (31.6)
Absent	4 (21.0)
Cervical stromal invasion	4 (21.1)
Lymph node involvement	3 (15.8)
Rectal and urinary bladder wall invasion	1 (5.3)
Involvement in the wall of the sigmoid colon	1 (5.3%)

On DWI

Bright areas were seen on trace images with corresponding dark areas on ADC mapping in all 19 cases (100%) of EC representing diffusion restriction (Table 2). The mean ADC value was found to be lower in involved myometrium as compared to non-involved myometrium (Table 3).

**Table 3:** A comparison of mean ADC and DTI parameters of our study with previous literature ADC = apparent diffusion coefficient, FA = fractional anisotropy, AD = axial diffusivity, MD mean diffusivity, IM = involved myometrium, and NM = non-involved myometrium. *P-value by the paired t-test.

Studies	ADC value (10^-3^mm^2^/s)		FA		λ_1_(AD) (10^-3^)		λ_2_ (10^-3^)		λ_3_ (10^-3^)		MD (10^-3^)	
	IM	NM	IM	NM	IM	NM	IM	NM	IM	NM	IM	NM
Tamai et al. [11]	0.88 ± 0.16	-	-	-	-	-	-	-	-	-	-	-
Kececi et al. [12]	0.94 ± 0.18	-	-	-	-	-	-	-	-	-	-	-
Bharwani et al. [13]	0.97 ± 0.31	1.50 ± 0.14	-	-	-	-	-	-	-	-	-	-
Toba et al. [14]	-	-	0.21 ± 0.05	0.44 ± 0.01	-	-	-	-	-	-	-	-
Yamada et al. [15]	-	-	0.299 ± 0.051	0.471 ± 0.091	0.977 ± 0.120	2.655 ± 0.203	-	-	-	-	0.836 ± 0.117	1.808 ± 0.121
Our study	0.64 ± 0.16	1.38 ± 0.20	0.14 ± 0.05	0.30 ± 0.09	1.28 ± 0.27	2.21 ± 0.32	1.02 ± 0.25	1.78 ± 0.23	0.79 ± 0.16	1.43 ± 0.22	1.03 ± 0.20	1.81 ± 0.23
P value in our study	0.001^*^		0.001^*^		0.001^*^		0.001^*^		0.001^*^		0.001^*^	

On DCE-MRI

Of the 19 cases studied, 18 (94.7%) had mild heterogeneous enhancement in arterial, equilibrium, and delayed phases. Differentiation between tumor and normal myometrium was found to be more apparent in the equilibrium phase.

Distribution of Myometrial Invasion on DCE-MRI

**Figure 1:**
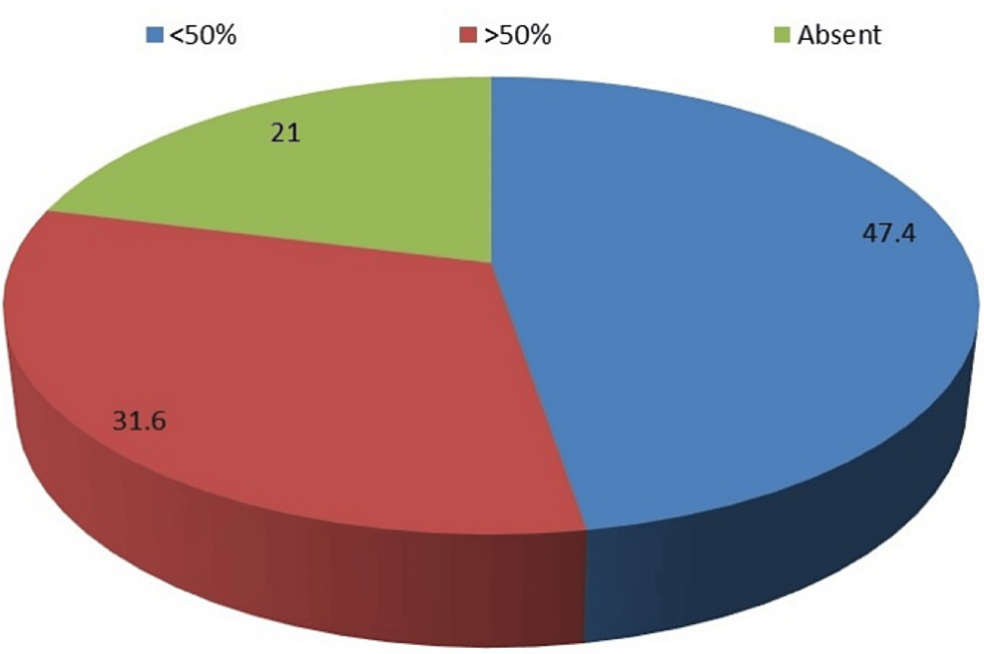
Distribution of myometrial infiltration on DCE-MRI DCE-MRI = dynamic contrast-enhanced MRI

Of the 19 cases studied, nine (47.4%) had < 50% of myometrial invasion, six (31.6%) had > 50%, and four (21%) had none (Figure 1).

Distribution of Involvement of Other Pelvic Structures on MRI

Four had (21.1%) cervical stromal invasion, one (5.3%) had involvement in the wall of the sigmoid colon, and one (5.3%) had rectal and urinary bladder wall infiltration. Three cases had (15.8%) pelvic lymph node involvement in our study population (Table 2).

On DTI

We assessed DTI parameters such as mean FA, AD, MD, and eigenvalues in our study. These parameters were lower in invaded myometrium as compared to non-involved myometrium and found to be statistically significant (Table 3).

Distribution of FIGO Staging on MRI

**Figure 2:**
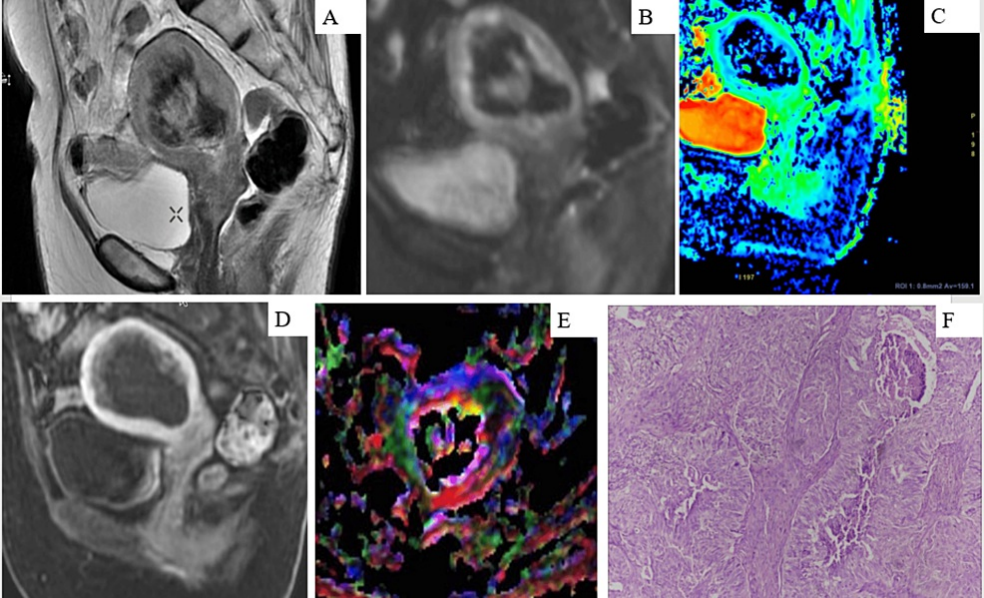
(A) Sagittal T2W image: an irregularly marginated heterogeneously hyperintense mass lesion in the endometrial cavity of the uterus. (B) Sagittal DWI: appearing areas with bright signal intensity on the trace image and corresponding dark areas on the ADC image (C), representing diffusion restriction. (D) The sagittal DCE-MRI image shows mild heterogeneous enhancement. (E) DTI mapping shows more than 50% myometrial involvement representing FIGO stage IB endometrial carcinoma. (F) The microphotograph showing a tumor arranged in a complex papillary pattern with a solid area comprising of squamous nodules displaying comedo necrosis consistent with endometrioid adenocarcinoma (H&E, x10) FIGO stage IB DWI = diffusion-weighted imaging, ADC = apparent diffusion coefficient, DCE-MRI = dynamic contrast-enhanced MRI, DTI = diffusion tensor imaging

**Figure 3:**
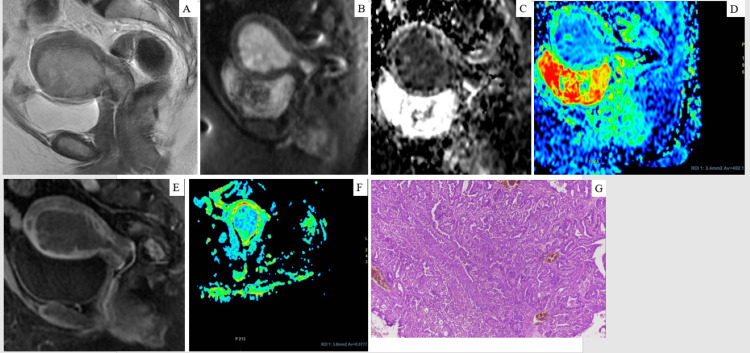
(A) Sagittal T2W image: a hyperintense mass lesion in the endometrial cavity of the uterus with extension into the endocervical canal and obliteration of normal cervical stroma representing invasion. (B) Sagittal DWI appears bright signal intensity on trace image. (C) ADC image shows corresponding dark representing diffusion restriction. (D) Measuring an ADC value with the ROI placed in the ADC map. (E) DCE-MRI image shows mild heterogeneous enhancement, including cervical stroma. (F) DTI mapping shows an endometrial lesion in the uterus. Findings are consistent with FIGO stage II endometrial carcinoma. (G) A tumour arranged in a complex papillaroid pattern with scant intervening stroma consistent with endometrioid adenocarcinoma (H&E, x10), FIGO stage II DWI = diffusion-weighted imaging, ADC = apparent diffusion coefficient, DCE-MRI = dynamic contrast-enhanced MRI, DTI = diffusion tensor imaging

Based on FIGO staging, out of 19 cases studied, 13 (68.4%) had FIGO stage IA, one (5.3%) had FIGO stage IB, two (10.5%) had FIGO stage II, one (5.3%) had FIGO stage IIIC, and two (10.5%) had FIGO stage IVA in the study group. A few examples are shown in Figures 2-3 (Figures 2A-2F, Figures 3A-3G).

Surgicopathological Staging in Comparison to Preoperative MRI-Based FIGO Staging

Out of 15 patients who underwent surgicopathological staging, we observed under-staging in stages IB and IVB, while in the rest of the cases, MRI-based FIGO staging accurately matched with surgicopathological staging (Table 4).

**Table 4:** Distribution of MRI staging according to surgicopathological staging among the case studies MRI = magnetic resonance imaging, n = study population, % = percentage, FIGO staging = International Federation of Gynecology and Obstetrics staging. *P-value<0.001 by the chi-square test.

	FIGO Staging on MRI												
	FIGO Stage IA		FIGO Stage IB		FIGO Stage II		FIGO Stage IIIC		FIGO Stage IVA		Total		P-value
Surgicopathological FIGO staging	n	%	n	%	n	%	n	%	n	%			
FIGO Stage IA	11	100.0	0	0.0	0	0.0	0	0.0	0	0.0	11	100.0	0.001^*^
FIGO Stage IB	1	50.0	1	50.0	0	0.0	0	0.0	0	0.0	2	100.0	
FIGO Stage II	0	0.0	0	0.0	1	100.0	0	0.0	0	0.0	1	100.0	
FIGO Stage III	0	0.0	0	0.0	0	0.0	0	0.0	0	0.0	0	0.0	
FIGO Stage IVA	0	0.0	0	0.0	0	0.0	0	0.0	0	0.0	0	0.0	
FIGO Stage IVB	0	0.0	0	0.0	0	0.0	0	0.0	1	100.0	1	100.0	
Total	12	80.0	1	6.7	1	6.7	0	0.0	1	6.7	15	100.0	

## Discussion

A total of 19 patients with histopathologically confirmed cases of EC underwent mpMRI and were included in our study, while 15 cases underwent surgicopathological staging. We found a significant correlation of mpMRI, mainly DCE-MRI, DTI, and DWI in the assessment and prognostication of EC.

Clinical presentation

Similar to previous studies, we found that EC was common in postmenopausal females mostly occurring after 50 years of age [16,17]. Shrivastava et al. revealed that the most common complaint in postmenopausal patients was bleeding per vagina, constituting 72%, while 16.7% of patients of the perimenopausal age group presented with irregular and heavy bleeding per vagina, similar to our findings [18]. Although nulliparity was considered a risk factor for EC by previous studies, our study showed more association with multiparous women [17,19]. This might be due to a small sample size and not representative of the total population.

Histopathologic findings

We encountered endometroid adenocarcinoma in the majority (78.9%) of the cases in concurrence with that reported by Shrivastava et al. (75%) and by Ghosh et al. (70.37%) [18,20].

MRI

Similar to previous studies, the heterogeneously hyperintense signal was most frequently observed on the T2W sequence in our study as compared to myometrium [5,11]. The heterogeneity is possibly due to areas of necrosis with a resultant slower rate of signal decay and increased T2 time.

DWI

DWI is based on microscopic water diffusivity in the tissue. High cellularity with reduced extracellular space limits the mobility of water molecules in cancerous cells causing diffusion restriction, as noted in our study. Moreover, the ADC value provides semi-quantification of diffusivity and is hence considered a better tool than subjective assessment. We found that involved myometrium had a lower ADC value as compared to non-involved myometrium similar to previous studies and was statistically significant [11-13] (Table 3). The difference in absolute value is possibly due to the use of a 3T scanner, minor difference in DWI parameters, and smaller sample size.

DCE-MRI

Owing to the different vascularity between tumor and myometrium, DCE-MRI can be an excellent tool to differentiate based on differential enhancement. The distinction of involved myometrium was more apparent in the equilibrium phase clearly distinguishing viable tumors from necrosis in our study, as seen in a previous study [9].

DTI

DTI parameters usually represent the density and orientation of fibers, which are distorted by infiltrating tumor cells. We found a significant reduction of these parameters such as mean FA, AD, MD, and eigenvalues in infiltrated myometrium as compared to normal, similar to previous studies [14,15,20]. However, a significant difference in their absolute values was seen [14,15] (Table 3). This might be due to a smaller sample size, 3T MRI, and an in-vivo study used in our study.

Surgico-pathological FIGO vs preoperative mpMRI staging

We found a statistically significant correlation between preoperative mpMRI-based FIGO staging with surgicopathological FIGO staging (Table 4). A significant under-staging of IB and IVB in our study may be due to the small sample size. However, the partial volume effect while placing a region of interest (ROI) may play a minor role in it.

We attempted to reach a cut-off value for differentiating myometrial invasion based on the ROC curve utilizing ADC and DTI parameters and found it to be statistically significant (Table 5).

**Table 5:** Cut-off values of various mpMRI parameters for the involved myometrium based on the ROC curve MRI = magnetic resonance imaging, ROC = receiver operating characteristic curve, AUC = area under curve, SE = standard error, CI = confidence interval

MRI Parameter	Optimal Diagnostic Cut-Off Based on ROC	AUC ± SE	95% CI of AUC	P-value
ADC value (10^-3^mm^2^/s)	0.94	0.999 ± 0.00	0.999 – 0.999	0.001^*^
FA	0.20	0.972 ± 0.029	0.915 – 0.999	0.001^*^
l1 (10^-3^)	1.65	0.986 ± 0.016	0.954 – 0.999	0.001^*^
l2 (10^-3^)	1.34	0.975 ± 0.026	0.925 – 0.999	0.001^*^
l3 (10^-3^)	1.05	0.991 ± 0.011	0.970 – 0.999	0.001^*^
MD (10^-3^)	1.35	0.996 ± 0.006	0.985 – 0.999	0.001^*^

Kececi et al. [12] and Bharwani et al. [13], in their studies, found the cut-off value for ADC to be 1.10x10^-3^ mm^2^/sec and 1.28x10^-3 ^mm^2^/sec, respectively, which was close to our finding. Similarly, the cut-off value for FA was found to be 0.356 by Zhang et al. was nearly close to our finding [21]. However, the absolute values for ADC and FA differ from previous studies possibly due to the smaller sample size, 3T scanner, and minor variation in scanning parameters. To the best of our knowledge, no literature could be found that used all these parameters in combination to differentiate tumoral infiltration into myometrium in EC. We assessed the diagnostic efficacy of these parameters in the form of sensitivity, specificity, positive predictive value, negative predictive value, and accuracy and found them to be significantly high (Table 6).

**Table 6:** Comparison of the diagnostic efficacy of various mpMRI parameters for the prediction of the involved myometrium in previous studies ADC = apparent diffusion coefficient, FA = fractional anisotropy, AD = axial diffusivity, MD = mean diffusivity, PPV = positive predictive value, NPV = negative predictive value

Parameters	Study name	Sensitivity	Specificity	PPV	NPV	Accuracy
ADC value (10^-3^mm^2^/s)	Our study	100.	100.0	100.0	100.0	100.0
	Kececi et al. [12]	85.7	92.8	97.2	68.4	-
	Bharwani et al. [13]	87	100	100	85.7	-
FA value	Our study	93.3	100.0	100.0	95.0	97.1
	Zhang et al. [21]	88.6	97.1	96.9	89.5	92.9
l1 value	Our study	93.3	100.0	100.0	95.0	97.1
	Zhang et al. [21]	77.78	76.92	-	-	-
l2 value	Our study	93.3	100.0	100.0	95.0	97.1
l3 value	Our study	93.3	100.0	100.0	95.0	97.1
MD value	Our study	93.3	100.0	100.0	95.0	97.1
	Zhang et al. [21]	77.78	92.31	-	-	-

In comparison to previous studies, these indices were found to be higher in our study. This might be due to the small sample size, the 3T scanner used, and minor variation in scanning parameters [17,18,21]. Himoto et al. revealed mp MRI was seen to increase the diagnostic accuracies moderately in detecting the spread of EC beyond endometrium in their multicentered retrospective study in concurrence with our findings [22].

The depth of myometrial invasion is a critical parameter in the assessment of EC. T2W can delineate the interruption of junctional zones. Since the junctional zone and myometrium are thinned out due to the involution of the uterus in postmenopausal women, it is difficult to assess using T2W only. Therefore, mpMRI can be used to improve diagnostic efficacy as revealed in our study. Moreover, lymph-nodal dissection is associated with significant morbidity and is usually done if > 50% of myometrial involvement, as seen in a previous study [17]. Thus, preoperative mpMRI can also be utilized for deciding nodal dissection.

Limitations

The small sample size in our study was the major limiting factor. Therefore, the cut-off values of various mpMRI parameters based on the ROC curve may not be accurate. A larger sample size is needed to support or refute the same. Secondly, the partial volume effect while placing ROIs cannot be entirely ruled out.

## Conclusions

Conventional MRI is a standard imaging investigation for endometrial lesions. However, its sensitivity and specificity to characterize endometrial lesions are relatively low. The addition of mpMRI (including DWI, DCE, and DTI) to the conventional technique has improved the diagnostic accuracy of EC, as revealed in our study.

We found a significant correlation between mpMRI imaging and histopathological findings in assessing myometrial infiltration. Therefore, in our opinion, mpMRI could improve the overall staging accuracy by accurately evaluating deep myometrial invasion and advanced-stage endometrial carcinoma. Moreover, lymph-nodal dissection is associated with significant morbidity and is usually indicated if > 50% of myometrial involvement is seen. Thus, preoperative mpMRI can also be utilized for deciding nodal dissection as well. The small sample size may not be a complete representative of the population, a major limiting factor in our study. Hence, further study in a larger sample may be carried out in the future to support or refute our findings.
